# Pyogenic liver abscess-caused *Klebsiella pneumoniae* in a tertiary hospital in China in 2017: implication of hypervirulent carbapenem-resistant strains

**DOI:** 10.1186/s12879-022-07648-0

**Published:** 2022-08-09

**Authors:** Hongchao Chen, Lanfang Fang, Wenjie Chen, Qing Yang, Dan Li, Dakang Hu, Jin Zhang

**Affiliations:** 1 Department of Laboratory Medicine, The First Affiliated Hospital, College of Medicine, Zhejiang University, Hangzhou, 310006 China; 2 Department of Infectious Diseases, The First Affiliated Hospital, College of Medicine, Zhejiang University, Hangzhou, 310006 China; 3 Department of Infectious Diseases, Huashan Hospital, Fudan University, Shanghai, 200040 China; 4 Institute of Antibiotics, Huashan Hospital, Fudan University, Shanghai, 200040 China; 5 Department of Laboratory Medicine, Taizhou Municipal Hospital, Taizhou, 318000 China

**Keywords:** *Klebsiella pneumoniae*, Pyogenic liver abscess, Multilocus sequence typing, Pulsed-field gel electrophoresis, *Galleria mellonella* lethality test

## Abstract

**Background:**

To investigate the epidemiology of *Klebsiella pneumoniae* (*K. pneumoniae*) inducing pyogenic liver abscess (PLA) in east China and the role of hypervirulent carbapenem-resistant *K. pneumoniae* (Hv-CRKP).

**Methods:**

Forty-three *K. pneumoniae* strains were collected from 43 patients with PLA at Hangzhou, China in 2017. Antimicrobial susceptibility tests, string test, multilocus sequence typing, pulsed-field gel electrophoresis, mobile genetic elements typing, regular PCR and sequencing, and *Galleria mellonella* (*G. mellonella*) lethality test were used to elucidate the epidemiology. Clinical data were collected.

**Results:**

*K. pneumoniae* strains with serotypes K1 and K2 accounted for 69.8%, which shared 46.5% and 23.3% respectively. *K. pneumoniae* strains with clonal group 23 were predominant with a rate of 34.9%. Such antimicrobials showed susceptible rates over 80.0%: cefuroxime, cefotaxime, gentamycin, ticarcillin/clavulanate, ceftazidime, cefoperazone/tazobactam, cefepime, aztreonam, imipenem, meropenem, amikacin, tobramycin, ciprofloxacin, levofloxacin, doxycycline, minocycline, tigecycline, chloramphenicol, and trimethoprim-sulfamethoxazole. PFGE dendrogram showed 29 clusters for the 43 *K. pneumoniae* strains. Three Hv-CRKP strains were confirmed by *G. mellonella* lethality test, showing a constituent ratio of 7.0% (3/43). Totally three deaths were found, presenting a rate of 7.0% (3/43). The three died patients were all infected with Hv-CRKP.

**Conclusions:**

K1 and K2 are the leading serotypes of *K. pneumoniae* causing PLA, which show highly divergent genetic backgrounds. Aminoglycosides, Generation 2^nd^ to 4^th^ cephalosporins, β-lactamase/β-lactamase inhibitors, carbapenems, fluoroquinolones are empirical choices. Hv-CRKP may confer an urgent challenge in the future.

**Supplementary Information:**

The online version contains supplementary material available at 10.1186/s12879-022-07648-0.

## Background

*Klebsiella pneumoniae* (*K. pneumoniae*) is a common bacterium that can cause various diseases in both immunocompromised and otherwise healthy individuals, such as pneumonia, bacteremia, urinary tract infection, and pyogenic liver abscess (PLA) [1]. PLA is a life-threatening disease that is frequently observed worldwide and, in particular, is endemic to East Asia, showing a morbidity rate of 15.45 per 100,000 person-years in 2011 and a mortality rate of 4.7–9.8% [2–4]. With respect to the causative agents, bacteria resulted in 75.0% of all types of liver abscess [5]; *K. pneumoniae* in particular accounted for 52.4–81.7% of bacteria that cause liver abscesses worldwide[4, 6–8].

Typically, *K. pneumoniae* strains that cause PLA were susceptible to antimicrobials, exception of intrinsic resistance [9, 10]. However, recent studies have revealed the emergence of carbapenem-resistant *K. pneumoniae* (CRKP) [4, 7, 11]. In the past three decades, cases of hypervirulent *K. pneumoniae* (HvKP), which is more virulent than classical *K. pneumoniae* (cKP), have been increasingly documented. HvKP could be differentiated from cKP via mouse or *Galleria mellonella* (*G. mellonella*) lethality tests [12, 13]. HvKP is more common in the Asian side of the Pacific Rim, but is emerging globally [14], causing a variety of invasive infections, such as lung abscess, PLA, and meningitis [15]. The hypercapsule of HvKP itself could mask the fimbriae and hamper conjugation. Nevertheless, the capsule of cKP is slim and often has an impaired immune response against exocellular mobile elements [16]. Thus, the increasing multidrug-resistant (MDR) HvKP evolves more often from cKP than HvKP because of the acquisition of mobile elements carrying virulence determinants, thereby resulting in nosocomial infections [12, 14]. Among the various strains, hypervirulent carbapenem-resistant *K. pneumoniae* (Hv-CRKP) has gained notoriety as a highly infectious pathogen due to an increase in the number of severe infections and the increasing scarcity of effective treatments, broadening the number of people susceptible to all types of infections [14, 17].

Our hospital once reported 45 *K. pneumoniae* strains giving rise to PLA, which were collected during 2008 and 2012 [9]. With the passage of 5–9 years, the traits of such strains change remarkably. Here, another 43 *K. pneumoniae* strains were analyzed for drug resistance, virulence genes, serotypes, sequence types (ST), pulsed-field gel electrophoresis (PFGE)-based phylogenetic analysis, mobile genetic element (MGE) types, and lethality. The role of Hv-CRKP in PLA is intensively discussed.

## Methods

### *K. pneumoniae* strains

All 43 *K. pneumoniae* strains were isolated from patients with PLA at Department of Infectious Diseases, the First Affiliated Hospital of Zhejiang University in 2017. The specimens included abscess, drainage, and puncture fluid. *K. pneumoniae* strains were confirmed using a matrix-assisted laser desorption/ionization time-of-flight mass spectrometry system (Bruker Daltonics Inc., Fremont, CA, USA). The strains were stored at − 80 °C prior to use. Standard strains *K. pneumoniae* ATCC 700603 and *Escherichia coli* ATCC 25922, purchased from the National Centre for Medical Culture Collection of China, were used as controls for strain identification and antimicrobial susceptibility testing (AST).

NTUH-K2044 (Accession number: AP006725.1) is a hypervirulent *K. pneumoniae* strain typed as K1 and was isolated from Department of Internal Medicine, National Taiwan University Hospital, Taipei, Taiwan [18]. HS11286 (Accession number: CP003200.1) is a hypovirulent *K. pneumoniae* typed as K47 and containing *bla*_KPC_ and was isolated from Department of Laboratory Medicine, Huashan Hospital, Fudan University, Shanghai, China [19]. Strains NTUH-K2044 and HS11286 were used as controls for string test and *G. mellonella* lethality test.

All the strains were non-repetitive. All patients were diagnosed with PLA based on pathological and imaging evidences (B-mode ultrasonography and computed X-ray tomography).

### Determination of hypermucoviscous phenotype

The hypermucoviscous phenotype was determined by “string test” as described previously [20]. Formation of a viscous string > 5 mm in length was considered as a positive phenotype.

### AST analyses

AST for the 43 *K. pneumoniae* strains was performed using a bioMérieux VITEK-2 analyzer (bioMérieux Co., Marcy-Etoile, France) and the Kirby-Bauer (K-B) method. The GN337 card included the antibiotics ticarcillin/clavulanate, piperacillin-tazobactam, ceftazidime, cefepime, cefoperazone/tazobactam, aztreonam, imipenem, meropenem, amikacin, tobramycin, ciprofloxacin, levofloxacin, doxycycline, minocycline, tigecycline, chloramphenicol, and trimethoprim-sulfamethoxazole. The K-B method included ampicillin, cefazolin, cefuroxime, cefotaxime, gentamycin, nitrofurantoin, and fosfomycin. AST results were elucidated based on the latest guidelines by the Clinical and Laboratory Standards Institute (CLSI; Pittsburgh, PA, USA), and the latest breakpoint by the European Committee on Antimicrobial Susceptibility Testing (EUCAST; Basel, Switzerland; for tigecycline). MDR strains were characterized as strains non-susceptible to three or more antimicrobial classes [21].

### Multilocus sequence typing (MLST)

DNA of all 43 *K. pneumoniae* strains was extracted using the QIAamp DNA mini kit (QIAGEN Co., Venlo, Netherlands). Seven housekeeping genes (*gapA, infB, mdh, pgi, phoE, rpoB,* and *tonB*) [22] were sequenced for STs of the 43 strains according to the *K. pneumoniae* MLST database given at the website (http://www.pasteur.fr/recherche/genopole/PF8/mlst/Kpneumoniae.html). The primers are shown in Additional file 1.

### Polymerase chain reaction (PCR) for serotypes, drug-resistance, and virulence genes

Serotypes (K1, K2, K5, K20, K54, and K57) [23, 24], drug-resistance genes (*bla*_KPC_, *bla*_KPC-2_, *bla*_CTX-M1_, *bla*_CTX-M2_, *bla*_CTX-M8_, *bla*_CTX-M9_, *bla*_OXA48_, *bla*_NDM_, *bla*_IMP_, and *bla*_SHV_) and virulence genes (*wzy-K1, wzx, wzc, allS, entB, irp2, ybtS, iroB, iroN, iucA, kfu, fimH, mrkD, wabG, uge, rmpA, rmpA2, c-rmpA, p-rmpA, p-rmpA2, terB, peg-344, peg-589* and *peg-1631*) [12, 25, 26] were all determined by regular PCR using an Applied Biosystems Veriti PCR system (ABI, San Ramon, CA, USA). The primers used are described in Additional file 1. Sequencing of *wzi* loci was also used to determine serotypes [27] by comparison with the database of Pasteur Institute (https://bigsdb.pasteur.fr/klebsiella/klebsiella.html).

### Definitions of putative HvKP, cKP, Hv-CRKP, and carbapenem-resistant HvKP strains

Hypercapsule-associated genes (*wzy-K1, c-rmpA, p-rmpA* and *p-rmpA2*) and siderophore genes (*entB, irp2, iroB,* and *iroN*) were included for screening HvKP and cKP. HvKP and cKP were putatively defined as described previously [28]. CRKP was defined as *K. pneumoniae* strains that are non-susceptible to imipenem or meropenem. Hv-CRKP was defined as CRKP (cKP) that acquires key virulence genes that confer hypervirulence. Carbapenem-resistant HvKP was defined as HvKP (serotypes K1, K2, K5, K10, K20, K25, K27, and K57) that acquires carbapenem resistance.

### MGE and PFGE analyses

MGE and PFGE analyses were both performed as the reference [29].

### *G. mellonella* lethality test

*G. mellonella* larvae were used to determine the lethality of *K. pneumoniae* strains [30]. *G. mellonella* larvae, weighing approximately 300 mg, were purchased from Tianjin Huiyude Biotech Company, Tianjin, China. Mid-log phase cultures of *K. pneumoniae* strains were washed with phosphate-buffered saline and further adjusted to a concentration of 1 × 10^7^ CFU/mL. Ten *G. mellonella* larvae were used per test. Survival analysis was done to compare the lethality of *K. pneumoniae* strains. All experiments were performed in triplicates.

### Statistical analysis

GraphPad Prism 8 (GraphPad Software Inc., USA) was used to perform Chi-square test, Fisher’s exact test and survival analysis. The value of *p* < 0.05 was regarded as statistically significant.

## Results

### General information of 43 *K. pneumoniae* strains

Characteristics of the 43 *K. pneumoniae* strains were shown in Table 1. Among the *K. pneumoniae* strains, the positive rate of “string test” was 27.9% (12/43). Serotypes K1 and K2 accounted for a total of 69.8% of the cases (30/43), with K1 and K2 accounting for 46.5% (20/43) and 23.3% (10/43), respectively. Clonal group (CG) 23 was predominant, with a share of 34.9% (15/43). Types D and M dominated MGE types with ratios of 62.8% (27/43) and 18.6% (8/43), respectively.

**Table 1:** Characteristics of the 43 *K. pneumoniae* strains

Strain	Specimen	String test	Capsule type	ST	MGE	CR	Outcome
H1	Abscess	−	ND	660	L	N	Survivor
H2	Drainage	+	K2	380	D	N	Survivor
H3	Puncture fluid	−	K1	23	D	N	Survivor
H4	Puncture fluid	+	K2	375	E	N	Survivor
H5	Puncture fluid	−	K1	23	D	N	Survivor
H6	Abscess	−	K20	420	D	N	Survivor
H8	Drainage	−	K1	2159	M	N	Survivor
H9	Puncture fluid	−	K1	CG23	D	N	Survivor
H10	Abscess	+	K1	23	D	N	Survivor
H11	Abscess	−	K1	23	M	N	Survivor
H12	Drainage	−	K1	1265	D	N	Survivor
H13	Abscess	−	K54	29	D	N	Survivor
H14	Abscess	−	K1	23	D	N	Survivor
H15	Puncture fluid	−	K64	11	A	Y	Survivor
H16	Abscess	−	K1	23	D	N	Survivor
H17	Abscess	+	K2	86	D	N	Survivor
H18	Abscess	−	ND	4060	M	N	Survivor
H19	Drainage	−	K1	700	D	N	Survivor
H20	Abscess	−	K54	29	D	N	Survivor
H21	Drainage	+	K2	65	D	N	Survivor
H22	Puncture fluid	+	K57	412	M	N	Survivor
H23	Puncture fluid	+	K2	380	D	N	Survivor
H24	Drainage	+	K2	65	D	N	Survivor
H25	Puncture fluid	−	ND	309	C	N	Survivor
H26	Puncture fluid	−	K2	65	M	N	Survivor
H27	Puncture fluid	−	K1	23	D	N	Survivor
H28	Puncture fluid	−	K1	23	D	N	Survivor
H29	Abscess	+	K1	23	D	N	Survivor
H30	Puncture fluid	+	K2	25	M	N	Survivor
H31	Puncture fluid	+	K2	375	E	N	Survivor
H32	Abscess	−	K1	700	D	N	Survivor
H33	Abscess	−	K57	592	M	N	Survivor
H34	Puncture fluid	−	K1	700	D	N	Survivor
H35	Puncture fluid	−	K1	23	D	N	Survivor
H36	Abscess	−	K16	660	G	Y	Non-survivor
H37	Drainage	+	K2	2165	M	N	Survivor
H38	Drainage	−	K16	660	G	Y	Non-survivor
H39	Abscess	−	K1	23	D	N	Survivor
H40	Abscess	−	K1	700	D	N	Survivor
H41	Abscess	−	K1	23	D	N	Survivor
H42	Drainage	−	K64	11	A	Y	Non-survivor
H44	Abscess	−	K1	23	D	N	Survivor
H45	Puncture fluid	−	K64	692	D	N	Survivor

− negative, + positive, *ND* not defined, *CG* clonal group, *ST* sequence type, *MGE* mobile genetic element, *CR* carbapenem-resistance, *N* No, *Y* yes

### Drug-resistance of 43 *K. pneumoniae* strains

Among the 24 kinds of antibiotics, the ones that showed susceptibility rates of over 80.0% included cefuroxime, cefotaxime, gentamycin, ticarcillin/clavulanate, ceftazidime, cefoperazone/tazobactam, cefepime, aztreonam, imipenem, meropenem, amikacin, tobramycin, ciprofloxacin, levofloxacin, doxycycline, minocycline, tigecycline, chloramphenicol, and trimethoprim-sulfamethoxazole according to Additional file 1.

### Prevalence of drug-resistance and virulence-related genes

**Fig. 1:**
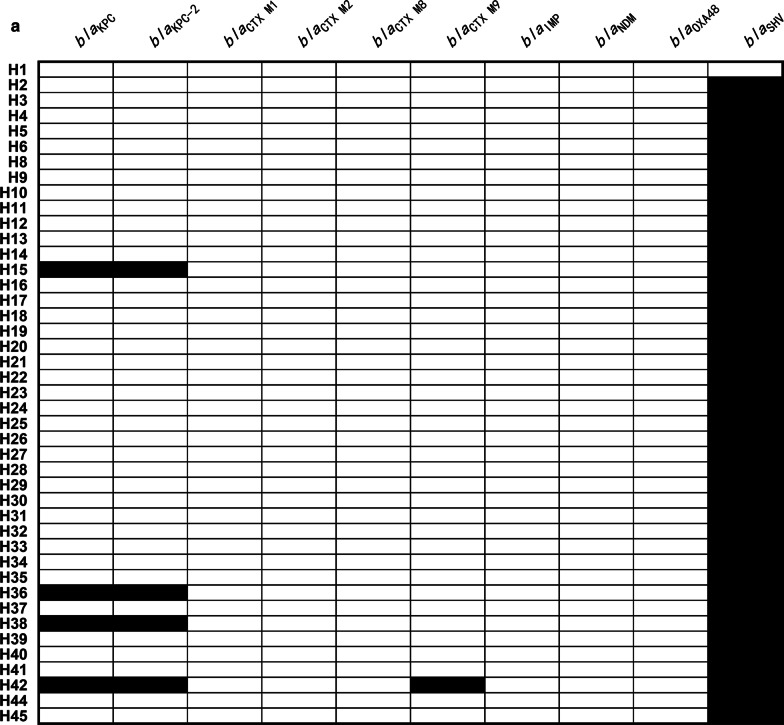

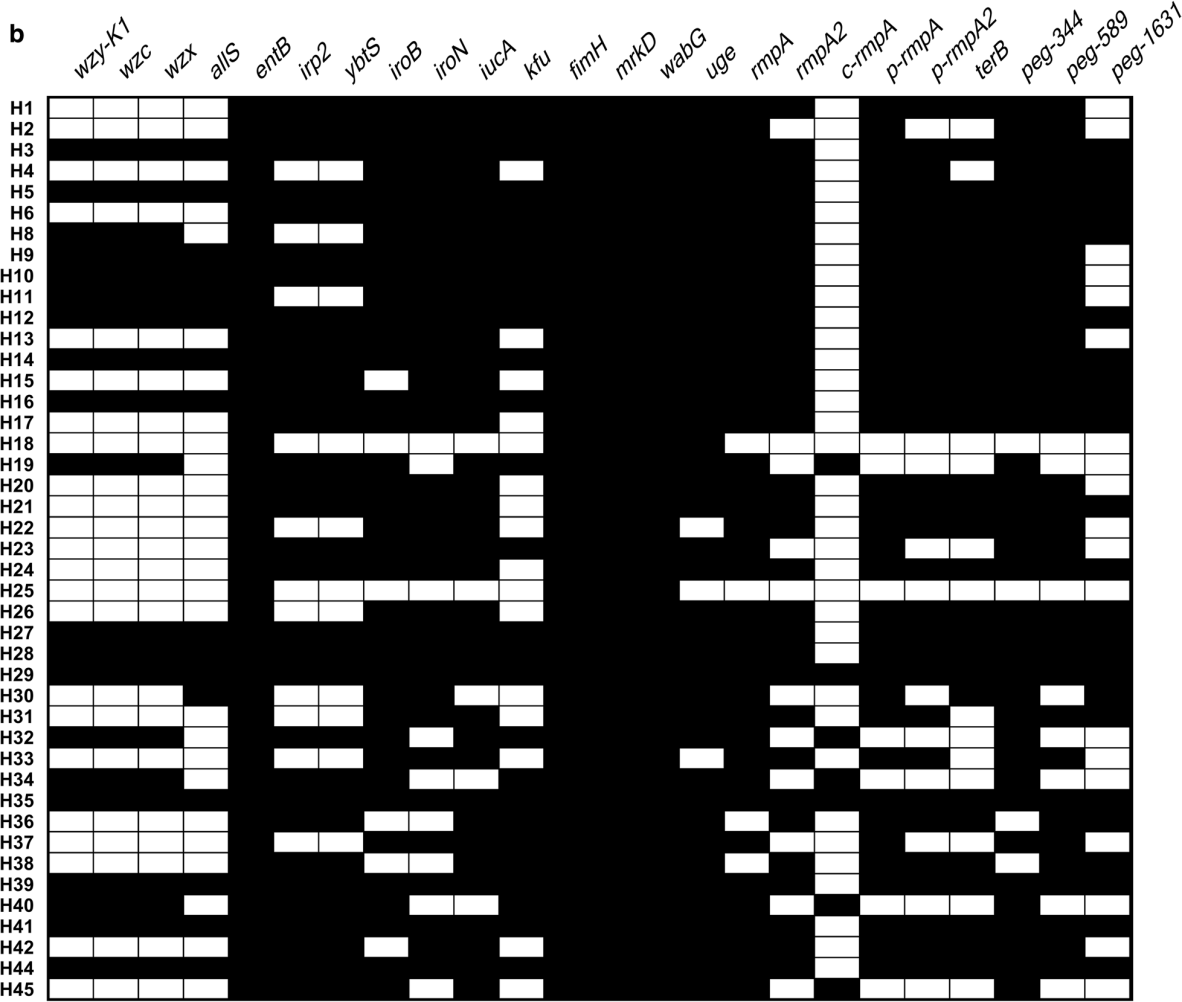
**a** Prevalence of ten drug-resistance genes; **b** Prevalence of twenty-four virulence-related genes. The presence of drug-resistance and virulence-related genes is represented by a black box, and the absence of others is represented by a white box. Ten *bla* genes for the 43 *K. pneumoniae* strains are shown in (**a**). Twenty-four virulence-related genes for the 43 *K. pneumoniae* strains are shown in (**b**)

As shown in Fig. 1a, *bla*_KPC_ and *bla*_KPC-2_ were positive in strains H15, H36, H38, and H42, showing a rate of 9.3% (4/43); *bla*_SHV_ was positive in all the strains except strain H1.

The detection rates of virulence genes varied remarkably: *c-rmpA* (7/43, 16.3%), *allS* (16/43, 37.2%), *wzy-K1*(20/43, 46.5%), *wzc* (20/43, 46.5%), *wzx* (20/43, 46.5%), *peg-1631* (24/43, 55.8%), *kfu* (27/43, 62.8%), *terB* (30/43, 69.8%), *irp2* (32/43, 74.4%), *ybts* (32/43, 74.4%), *rmpA2* (32/43, 74.4%), *p-rmpA2* (32/43, 74.4%), *iroN* (34/43, 79.1%), *peg-589* (35/43, 81.4%), *iucA* (36/43, 83.7%), *p-rmpA* (36/43, 83.7%), *iroB* (37/43, 86.0%), *rmpA* (39/43, 90.7%), *peg-344* (39/43, 90.7%), *uge* (40/43, 93.0%), *entB* (43/43, 100.0%), *fimH* (43/43, 100.0%), *mrkD* (43/43, 100.0%), and *wabG* (43/43, 100.0%). The following virulence genes represent certain siderophores: *entB*, enterobactin; *irp2* and *ybtS*, yersiniabactin; *iroB* and *iroN*, salmochelin; *iucA*, aerobactin. In addition, *fimH* and *mrkD* represent type 1 and type 3 fimbriae, respectively, and *wabG* and *uge* represent lipopolysaccharides. As shown in Fig. 1b, *wzx*, *wzc*, and *allS* coincided well with *wzy-K1* at rates of 100.0%, 100.0%, and 75.0%, respectively. The positive rates of *entB* and *irp2* were different: 100% (43/43) vs. 74.4% (32/43) (*p* = 0.0005). The positive rate of putative HvKP was 95.3% (41/43), except for H18 and H25. As shown in Fig. 1a, b, strains H15, H36, H38, and H42 were all putative Hv-CRKP.

### PFGE dendrograms

**Fig. 2:**
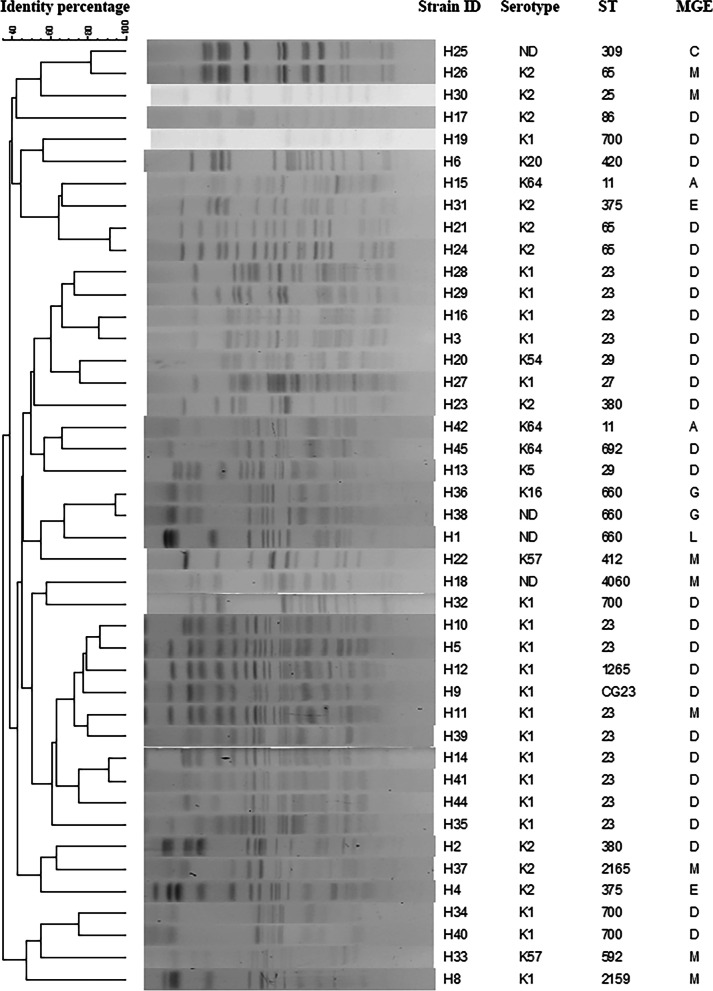
PFGE dendrogram of the 43 K*. pneumoniae* strains. ST, sequence type; MGE, mobile genetic element. CG, clonal group. Genetic relationships among the 43 K*. pneumoniae* strains are shown in Fig. 2. In addition, the serotype, sequence type and mobile genetic element type of each strain are together indicated

Figure 2 shows a total of 29 clusters, indicating highly divergent origins for the 43 *K. pneumoniae* strains. However, putative Hv-CRKP strains H36 and H38 presented the same background for both PFGE dendrogram and MGE type, showing that they belonged to the same clone. The other putative Hv-CRKP strains, H15 and H42, belonged to different clones. Therefore, all the four putative Hv-CRKP strains originated from three distinct clones.

### Clinical traits of four patients infected with putative Hv-CRKP

The demographic and clinical traits of the four patients who were infected with putative Hv-CRKP strains are shown in Table 2. Patient 15 was with several underlying conditions, no severe syndromes, had underwent surgery, and eventually survived. The other 3 patients were all diagnosed with several underlying diseases, had surgeries, and eventually died.

**Table 2:** Clinical characteristics of four patients infected with putative Hv-CRKP

	Patient 15 (H15)	Patient 36 (H36)	Patient 38 (H38)	Patient 42 (H42)
Clinical characteristics				
City	Huangshan	Hangzhou	Hangzhou	Huzhou
Ward	Department of Infectious Diseases	Department of Infectious Diseases	Anorectal Surgery, Intensive Care Unit, Department of Infectious Diseases	Department of Infectious Diseases
Underlying conditions	Septicemia, pneumonia, post-cholecystectomy, hepatic syst, cervical abscess	Dysfunction of liver, hepatic failure, Sjogren's syndrome, cholecystolithiasis, pulmonary infection, cervical erosion	General peritonitis, enterobrosis, intestinal obstruction, postoperative colon cancer, alcoholic liver disease, hypertension, diabetes mellitus, splenomegaly	Urinary tract infection, brain contusion, intracranial hemorrhage, epilepsia, hypertension
Invasive procedures				
Mechanical ventilation	No	Yes	Yes	No
Drainage catheters	No	Abdominal drainage tube	Abdominal drainage tube	No
Surgery	No	Yes	Yes	Yes
Date of admission	2017-11-3	2017-5-17	2017-5-31	2017-8-2
Date of specimen collection	2017-11-17	2017-7-21	2017-7-20	2017-9-25
Infection type	Pyogenic liver abscess	Abdominal infection	Peritonitis	Urinary tract infection, blood stream infection
Specimen type	Puncture fluid	Abscess	Drainage	Drainage
Prior treatment with broad spectrum antibiotics > 7 days within 2 months	Yes	Yes	No	No
Hospitalization within 90 days	No	No	No	No
Clinical presentations				
Temperature (T_max_) (℃)	40.4	38.4	38.3	39.1
Septic shock	No	Yes	Yes	No
WBC (10^9^/L)	13.6	9.6	26.3	21.4
C-reactive protein (mg/L)	108.7	203	60.38	124
PaO_2_/FiO_2_		96.8	89%	
ALT (IU/L)	89	139	55	43
LDH (U/L)	215	270	284	
Cr (μmol/L)	34	35	113	60
Antimicrobials used after isolation of *K. pneumoniae*	Tigecycline, meropenem	Tigecycline, amikacin	Piperacillin/tazobactam, meropenem	Tigecycline, meropenem, polymyxin
Clinical outcomes				
Length of stay (days)	14	64	50	53
Days of mechanical ventilation	0	9	15	0
Duration of ICU stay (days)	0	21	30	0
Outcome	Survived	Died	Died	Died

*WBC* white blood cells, *PaO*_*2*_*/FiO*_*2*_ alveolar oxygen partial pressure/fraction of inspiration oxygen, *IU* international unit, *ALT* alanine aminotransferase, *LDH* lactic dehydrogenase, *Cr* creatinine, *ICU* intensive care unit

### *G. mellonella *lethality test

**Fig. 3:**
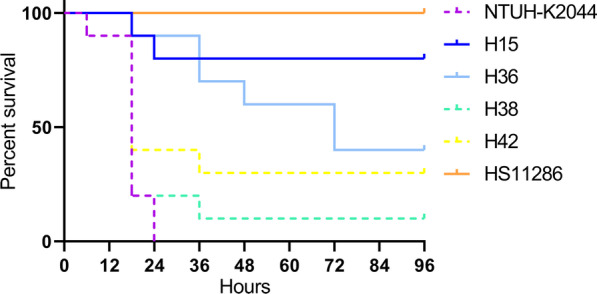
Survival curves of *G. mellonella* infected by *K. pneumoniae*. The percent survival of *G. mellonella* is shown in Fig. 3, which was injected with 0.1 mL of *K. pneumoniae* suspension at the concentration of 1 × 10^7^ CFU/mL. *CFU* colony forming unit

The four putative Hv-CRKP strains (H15, H36, H38, and H42) were analyzed for their lethality using the *G. mellonella* model. Log-rank (Mantel-Cox) test showed significant differences among six groups: χ^2^ = 40.5688 and *p* < 0.0001 (Fig. 3). Figure 3 also shows no significant difference among NTUH-K2044, H38, and H42 (χ^2^ = 5.0659 and *p* = 0.0794), and HS11286 and H15 (χ^2^ = 2.1096 and *p* = 0.1464). However, H36 was significantly different from NTUH-K2044 (χ^2^ = 16.4627 and *p* < 0.0001) and HS11286 (χ^2^ = 8.2092 and *p* = 0.0042). The overall survival rate of *G. mellonella* injected with H36 was 40.0%. Therefore, H36, H38, and H42 were denoted as Hv-CRKP, and H15 was confirmed as cKP.

## Discussion

We analyzed 43 *K. pneumoniae* strains that induced PLA, disclosed their molecular epidemiological status, and explored the emerging trend of Hv-CRKP strains in causing PLA. Serotypes K1 and K2, clonal group 23, and MGE types D and M predominated the 43 strains with rates of 69.8%, 34.9%, and 81.4%, respectively. According to the susceptibilities of the 43 strains, aminoglycosides, generation 2^nd^-4^th^ cephalosporins, β-lactamase/β-lactamase inhibitors, carbapenems, and fluoroquinolones could still be appropriate, alternative, and empirical treatment choices. The PFGE dendrogram confirmed the highly divergent origins of the 43 strains. These findings were in line with previous reports [9, 11]. However, in comparison with data obtained in a previous study [9], the incidence of serotype K1 decreased (χ^2^ = 4.5186 and *p* = 0.0335) and that of serotype K2 was equal (χ^2^ = 0.1377 and *p* = 0.7106), indicating a new trend in PLA.

*K. pneumoniae* can harbor many factors, such as capsule, siderophore, exopolysaccharide, fimbriae, of which the first three could determine whether it is hypervirulent or not [1, 14, 18]. In this study, 24 virulence-associated genes were identified. As shown in Fig. 1b, *wzx*, *wzc*, and *allS* coincided well with *wzy-K1* with rates of 100.0%, 100.0%, and 75.0%, respectively, indicating that these three genes are associated with serotype K1. Although enterobactin and yersiniabactin are “basic” siderophores for *K. pneumoniae*, the positivity rate of *entB* was higher than that of *irp2* (*p* = 0.0005). There are several markers for HvKP, such as string test, *rmpA*, and *peg-344* [14, 26]. The positive rate of string test among HvKP ranged from 27.9% to 90.7% based on different criteria. The poor positive rate of string test in this study declared its antiquation. There is an inevitable bias if only 1–3 genes are termed as markers of HvKP. Detection of *c-rmpA* in combination with *p-rmpA* equaled that of *rmpA* only: 41/43 vs. 39/43 (*p* = 0.6761). Intriguingly, *c-rmpA* was only present in serotype K1 of *K. pneumoniae* with positivity rates of 16.3% (7/43) in 43 strains and 35.0% (7/20) in K1 *K. pneumoniae,* indicating *that rmpA* is transposed into the chromosome of K1 *K. pneumoniae* more readily than other serotypes of *K. pneumoniae*. Although *peg-344* is not an exact virulence gene [31], it served as a better indicator of HvKP than *peg-1631*: 39/43 vs. 24/43 (χ^2^ = 15.0938 and *p* = 0.0001) and equaled *peg-589*: 39/43 vs. 35/43 (χ^2^ = 1.5496 and *p* = 0.2132).

In this study, three strains (H36, H38, and H42) were confirmed to be Hv-CRKP with a positivity rate of 7.0% (3/43) due to *bla*_KPC-2_, which was the predominant *bla*_KPC_ in China [32]. According to ST (ST11 and ST660) and MGE (A and G) types, these three strains belonged to two clusters, suggesting their different origins: ST11-K64 strain versus ST660-K16, which is different from what was observed in a previous study [8]. The rate of *bla*_KPC-2_-producing ST11 in Hv-CRKP was 33.3%, similar to that reported previously [8]. However, ST660 also shared a 66.7% rate, which was zero in the previous study [8]. *K. pneumoniae* strains in the previous study [8] were collected from 15 centers located in 11 Chinese cities, and the data in it reflected the general prevalence of Hv-CRKP that causes PLA in mainland China from 2012 to 2016. All three strains were MDR (Additional file 1) [21], which brought therapeutic challenges clinically. Although there are several methods for treating PLA, such as various drainage techniques, antimicrobials are still essential.

The first Hv-CRKP in mainland China emerged in 2013 [13]. Thereafter, the rate of Hv-CRKP was thought to increase gradually to 7.4%-15.0% [33]. Another five Hv-CRKP strains were reported in 2018 [12], which showed extremely high virulence and resulted in 100.0% deaths; they were the same clone and belonged to ST11. For virulence, they had three siderophores (enterobactin, yersiniabactin, and aerobactin) and *rmpA2*. In our study, H42 possessed four siderophores (enterobactin, yersiniabactin, salmochelin, and aerobactin), *rmpA,* and *rmpA2*, whereas H36 and H38 harbored three siderophores (enterobactin, yersiniabactin, and aerobactin) and *rmpA2*. H36, H38, and H42 also caused deaths, which is of great concern. Furthermore, the 3 deaths were the only ones in this study, indicating the important role of Hv-CRKP in PLA. Hv-CRKP, armed with its hypercapsule, could effectively resist the phagocytosis of leukocytes and enable systemic tissue invasion as a “Trojan horse”, resulting in thrombophlebitis, meningitis, etc. [14]. With the increasing incidence of metastatic *K. pneumoniae* meningitis, secondary to PLA, *K. pneumoniae* has become the leading pathogen of adult community-acquired bacterial meningitis instead of *Streptococcus pneumoniae* in Taiwan [34]. Due to extreme drug resistance and hypervirulence, Hv-CRKP may be a notable superbug in the future.

This study had some limitations. First, the sample size was small. Second, H36 and H38 showed different virulence, although PFGE confirmed the same origin. It may result from some slight differences of the genomes between H36 and H38 for virulence is the overall outcome of a series of virulence genes.

Taken together, we report the molecular characteristics of 43 different *K. pneumoniae* strains that caused PLA in 2017, which differed from the strains described in a previous study conducted between 2008 and 2012 in a tertiary hospital in East China. Our study highlights the imperative need to note the role of Hv-CRKP in PLA.

## Supplementary Information

**Additional file 1. **Primers, Mobile genetic element (MGE), Drug-resistance, Sequence type (ST), String test.

## Data Availability

The datasets analyzed for this study can be found in Additional file 1. The sequencing raw data are deposited in Sequence Read Archive (SRA) (Accession Number PRJNA851403).

## Abbreviations

*K.** pneumoniae*: *Klebsiella pneumoniae*
PLA: Pyogenic liver abscess
Hv-CRKP: Hypervirulent carbapenem-resistant *Klebsiella pneumoniae*
*G**.**mellonella*: *Galleria* *mellonella*
CRKP: Carbapenem-resistant *Klebsiella* *pneumoniae*
*bla*_KPC_: Beta-lactamase *Klebsiella pneumoniae* carbapenemase gene
*bla*_NDM_: Beta-lactamase New Delhi metallo-β-lactamase gene
*bla*_OXA_-48: Oxacillinase-48 gene
HvKP: Hypervirulent *Klebsiella pneumoniae*
cKP: Classical *Klebsiella pneumoniae*
MDR: Multidrug-resistant
ST: Sequence type
PFGE: Pulsed-field gel electrophoresis
MGE: Mobile genetic element
